# Temporomandibular disorders in individuals with Marfan syndrome: an exploratory analysis

**DOI:** 10.1186/s13005-024-00427-z

**Published:** 2024-04-24

**Authors:** Termeh Jenabzadeh, Lauren Bohner, Jeanette Köppe, Johannes Kleinheinz, Marcel Hanisch, Ole Oelerich

**Affiliations:** 1 Department of Oral and Maxillofacial Surgery, University Hospital Münster, 48149 Münster, Germany; 2 Institute of Biostatistics and Clinical Research, University of Münster, Schmeddingstraße 56, D-48149 Münster, Germany; 3 Department of Prosthodontics, University Hospital Münster, D-48149 Münster, Germany

**Keywords:** Marfan syndrome, Temporomandibular disorders, Chronic pain, Psychological impairment, Oral health

## Abstract

**Background:**

This study aims to analyze to what extent patients with Marfan syndrome (MFS) are affected by temporomandibular disorders (TMD) and its impact on oral health-related quality of life (OHRQoL). To collect data, an online questionnaire was created to recruit participants from Germany, Austria, and Switzerland through social media and support groups. The questionnaire consists of free-text questions, the German versions of the Oral Health Impact Profile (OHIP-G14), the Depression Anxiety Stress Scale (DASS), and the Graded Chronic Pain Status (GCPS).

**Results:**

A total of 76 participants with diagnosed MFS were included. Of these, 65.8% showed TMD symptoms, the most common being pain or stiffness of the masticatory muscles in the jaw angle (50.0%). Only 14.5% of the participants were already diagnosed with TMD. Of the participants with an increased likelihood of a depression disorder, 76.9% showed TMD symptoms. Of those with a critical score for an anxiety disorder, 90.9% showed TMD symptoms. 73.3% of participants with TMD symptoms reached the critical score for a stress disorder. TMD symptoms were associated with a higher risk for chronic pain. In the median, participants with TMD showed statistically notably higher OHIP-G14 scores than participants without TMD (11.5 [IQR 17] vs. 1 [IQR 3] points, *p* ≤ 0.001).

**Conclusion:**

TMD symptoms had a noticeable impact on OHRQoL in patients with MFS, i.e., chronic pain and psychological impairment. TMD seems underdiagnosed, and more research is needed to prevent the associated chronification of pain and psychological burden to improve the OHRQoL.

**Supplementary Information:**

The online version contains supplementary material available at 10.1186/s13005-024-00427-z.

## Background

Marfan syndrome (MFS) is a rare disease often associated with the involvement of the oral and maxillofacial region [1]. It is an autosomal dominant inherited connective tissue disorder, with an incidence of 1 in 10.000 cases for both sexes [2]. The syndrome is based on a mutation in the fibrillin-1 gene (FBN1) on chromosome 15q21 [1]. FBN1 encodes the protein fibrillin-1, an essential component of microfibrils in the extracellular matrix, both in elastic and non-elastic connective tissue [3, 4]. Accordingly, mutations are associated with tissue weakness, increased signaling of transforming growth factor β, and loss of cell-matrix interactions, ultimately leading to the various phenotypic manifestations of MFS [5].

In addition to general complications affecting the cardiovascular and musculoskeletal system, eyes, or lungs, MFS is associated with multiple oral manifestations [2, 6]. These include a narrow and high palate, maxillary and mandibular retrognathia, general malocclusion, crowding of the teeth, irregularities in the number of teeth, and TMD [6–8].

TMD comprises a group of disorders affecting the temporomandibular joint and surrounding structures [9]. Symptoms may include limited or painful jaw movement, neck pain, and cracking or rubbing of the temporomandibular joint(9). The increased presence of TMD could be the hypermobility of the joints, which is one of the common MFS symptoms. Previous studies in patients with generalized joint hypermobility (GJH) had demonstrated an increased incidence of non-painful subtypes of TMD [10]. In a GJH cohort (consisting of MFS and Ehlers-Danlos Syndrome (EDS) participants), an association between GJH and TMD was also demonstrated, showing myofascial pain in 69% of participants [11].

Previous studies have shown that both the presence of TMD and a diagnosis of Marfan syndrome can negatively affect OHRQoL. The TMJ pain, limited jaw mobility and other symptoms caused by TMD can often lead to physical and psychosocial impairment which can significantly affect the quality of life of those affected [12, 13]. In Marfan syndrome, oral symptoms can also have a negative impact on oral health and quality of life, leading to pain [6]. It is important to understand the specific effects of these two conditions on the OHRQoL in order to develop appropriate treatment strategies that can not only alleviate symptoms but also improve the quality of life of those affected. No studies have investigated the possible correlation between MFS, TMD, and oral health-related quality of life (OHRQoL). Only information about the prevalence of signs and symptoms of TMD in persons with MFS are available [14]. Accordingly, this explorative study aims to analyze to what extent persons with MFS are affected by TMD and how this impacts their quality of life.

## Materials and methods

### Recruitment and study design

An online questionnaire was used to collect data from participants. At the start, the participants were informed about the study contents and had to provide their consent to participate. It was available to participants from Germany, Austria, and Switzerland. Participants were recruited with the help of the Marfan Hilfe (Germany) e.V. (support group; NGO) by publishing a link to the questionnaire on their website. Additionally, the questionnaire was shared in different social media groups where people with the disease come together and exchange their experiences.

### Eligibility criteria

The requirements to participate were a minimum age of 18 years and the presence of MFS. The diagnosis had to be confirmed clinically or human genetically (or both). Only questionnaires in which all free-text questions were answered were included. In cases where data were missing from the validated questionnaires, these questionnaires were not regarded during the analysis. In the case of minor typographical mistakes, the questionnaires were included for evaluation if the answer could be inferred with certainty from the context. Those cases were resolved by discussion by two independent authors (T.J. and O.O).

### Data collection

The Data were collected over two months (June 2022-July 2022). A questionnaire consisting of free-text questions related to general participant information, oral health, and TMD symptoms was developed. A translated version of the questionnaire can be found in Supplementary File 1. Additionally, three validated questionnaires were used, the German short form of the Oral Health Impact Profile (OHIP-G14) [15], the German version of the Depression Anxiety Stress Scale (DASS) [16], and the German version of the Graded Chronic Pain Status, “Graduierung chronischer Schmerzen (GCS) [17, 18].

#### Evaluation of oral health and symptoms

Oral health and symptoms were recorded using free-text questions and selectable choices. Participants were asked how many teeth they had already lost and how many times they had been to the dentist in the previous 12 months. Participants could also tick the oral symptoms that applied to them, with multiple answers possible. The possible answers were absence or reduced effect of local anesthesia, pain of the masticatory muscles, parodontitis, hypodontia, disproportion of upper and lower jaw, cleft lip and palate, high palate, shape anomaly of the teeth, malformation of the tooth structure, dislocation of the temporomandibular joint and no oral involvement (see Supplementary File 1).

#### TMD-like symptoms

As the study was not accompanied by a clinical examination, it was not possible to categorize participants with or without TMD on the basis of Diagnostic Criteria for Temporomandibular Disorders (DC/TMD) [19]. However, in order to provide a subdivision of participants with and without TMD-like symptoms, two groups were formed. One group consisted of participants who reported that at least one movement of the lower jaw (opening, closing, sideways movement or chewing) was associated with pain or restriction, or in whom at least one masticatory muscle group (cheek, jaw angle, temple) was tense or painful. Participants who had already been diagnosed with TMD were also included in this group. The other group consisted of participants who had none of the above symptoms and had not been diagnosed with TMD.

#### Evaluation of OHRQoL

To evaluate the OHRQoL, the OHIP-G14 was used [15]. It consists of 14 questions and provides an overview of the psychosocial impairment of oral health. The questions are answered with a frequency score (“never” = 0, “hardly ever” = 1, “once in a while” = 2, “often” = 3, “very often” = 4). According to current recommendations, the OHIP-G14 was scored according to the four OHRQoL dimensions Oral Function, Orofacial Pain, Orofacial Appearance, and Psychosocial Impact [20]. For this purpose, only Physical Disability, Physical Pain, Psychological Discomfort, and Handicap domain scores were calculated and interpreted as Oral Function, Orofacial Pain, Orofacial Appearance, and Psychosocial Impact scores, respectively [20]. As each domain consists of two items, each with a possible score between 0 and 4, the dimensional scores could reach from 0 to 8 points, respectively. Additionally, a summary score of all items was calculated.

#### Psychological impairment

To evaluate the psychological impairment, the DASS was used [16]. The questionnaire consists of 21 questions, of which seven items are assigned to the three scales depression, anxiety, and stress. The answer indicates how much a statement applies or does not apply (“Did not apply to me at all - never” = 0, “Applied to me to some extent - sometimes” = 1, “Applied to me to a considerable extent - quite often” = 2, “Applied to me very much - most of the time” = 3). The scores are added separately for each of the three scales. The cut-off value for depression (increased likelihood of a depressive disorder) is 10, for anxiety, a cut-off value of 6, and 10 for stress.

#### Chronic pain graduation

For grading the level of chronic pain, the GCS was used [17, 18]. The questionnaire consists of seven questions, four referring to pain-related impairments and three to pain intensity. Responses are scored on a scale ranging from 0 to 10 (0 = no pain, 10 = pain could not be worse, or 0 = no impairment, 10 = you were unable to do anything) and a specification of days on which the everyday activities could not be performed in the last six months due to pain (question 1). For the evaluation, only questions 1, 5, 6, and 7 are to be considered, and the numbers given in the questions are converted into impairment points. An evaluation of questions 2, 3, and 4 (characteristic pain intensity) is only necessary if the sum of the calculated impairment points (from questions 1, 5, 6, and 7) is less than 3. Ultimately, the impairment points are categorized into grades 0–4. Grade 1 is classified as “low impairment - low pain intensity”, grade 2 as “low impairment - high pain intensity”, grade 3 as “severe impairment - moderate limitation” and grade 4 as “severe impairment - high limitation”. Grade 0 can be considered as “no pain”. Grades 1 and 2 are deemed persistent functional pain, and grades 3 and 4 are considered dysfunctional chronic pain.

### Statistic methods

The study was conducted to be fully explorative. Therefore, no power analysis was done a priori, and all results were interpreted as hypothesis generating.

To evaluate differences between participants with and without TMD symptoms, categorical variables (age, sex, country) were analyzed using a Chi-square Test. In addition, continuous variables (time of diagnosis, time between first symptoms and diagnosis) were analyzed using a Mann-Whitney-U Test. Differences in DASS and GCS scores between the two groups were analyzed using a Chi-square Test. OHRQoL and the four dimensions of OHRQoL were analyzed using a Mann-Whitney-U test. Differences between the levels of chronic pain on OHRQoL were tested via Kruskal-Wallis Test. The impact of psychological impairment on OHRQoL was analyzed using Mann-Whitney-U Test. All tests were performed at a significance level of α = 5%. Statistical analyses were performed using IBM SPSS Statistics for Mac, Version 28.0.1.0 (IBM Corp., Armonk, NY, USA) and graphics were designed using RStudio Version 2022.07.1 + 554 (RStudio PBC, Boston, MA, USA).

## Results

### Participants

In total, 81 individuals participated in the study. Five people were excluded as they were not eligible for the study. Four participants did not meet the age requirement, and one questionnaire was filled out incorrectly. A total of 76 questionnaires were included, of which 61 (80.3%) were female and 15 (19.7%) were male participants. 94.7% of those come from Germany, 3.9% from Austria, and 1.3% from Switzerland. The median age was 47 years (Interquartile range (IQR) 16), with the youngest being 21 and the oldest being 70. Detailed information on the participants depending on whether TMD symptoms are present can be found in Table 1.

### Diagnosis, diagnosis age, and diagnosis period

The majority of the patients were diagnosed both human genetically and clinically (63,2%), 13 (17,1%) patients were diagnosed only human genetically, and 15 (19,7%) only clinically. At the time of diagnosis, the median age was 26 (IQR 27), ranging from zero to a maximum of 56 years. The median time between the appearance of the first symptoms and the diagnosis of the MFS was six years (IQR 23), ranging from zero to a maximum of 50 years.

**Table 1:** General participants information temporomandibular disorders (TMD) symptoms

	n	Median	Range	TMD symptoms(*n* = 50)	no TMD symptoms (*n* = 26)	*p*-value
Age^1^ (IQR)		47 (16)	21–70	45 (18)	48.5 (17)	0.42
Sex (%)						0.019
male	15(19.7%)			6 (12.0%)	9 (34.6%)	
female	61(80.3%)			44 (88.0%)	17 (65.4%)	
Country (%)						0.767
Germany	72(94.7%)			47 (94.0%)	25 (96.2%)	
Austria	3(3.9%)			2 (4.0%)	1 (3.8%)	
Switzerland	1(1.3%)			1 (2.0%)	0 (0.0%)	
Time of diagnosis^1^ (IQR)		26 (27)	0–56	24.5 (27)	29.5 (29)	0.538
Time between first symptoms and diagnosis^1^ (IQR)		6 (23)	0–50	8 (20)	5.5 (27)	0.580

^1^-in years

### Oral symptoms

**Fig. 1:**
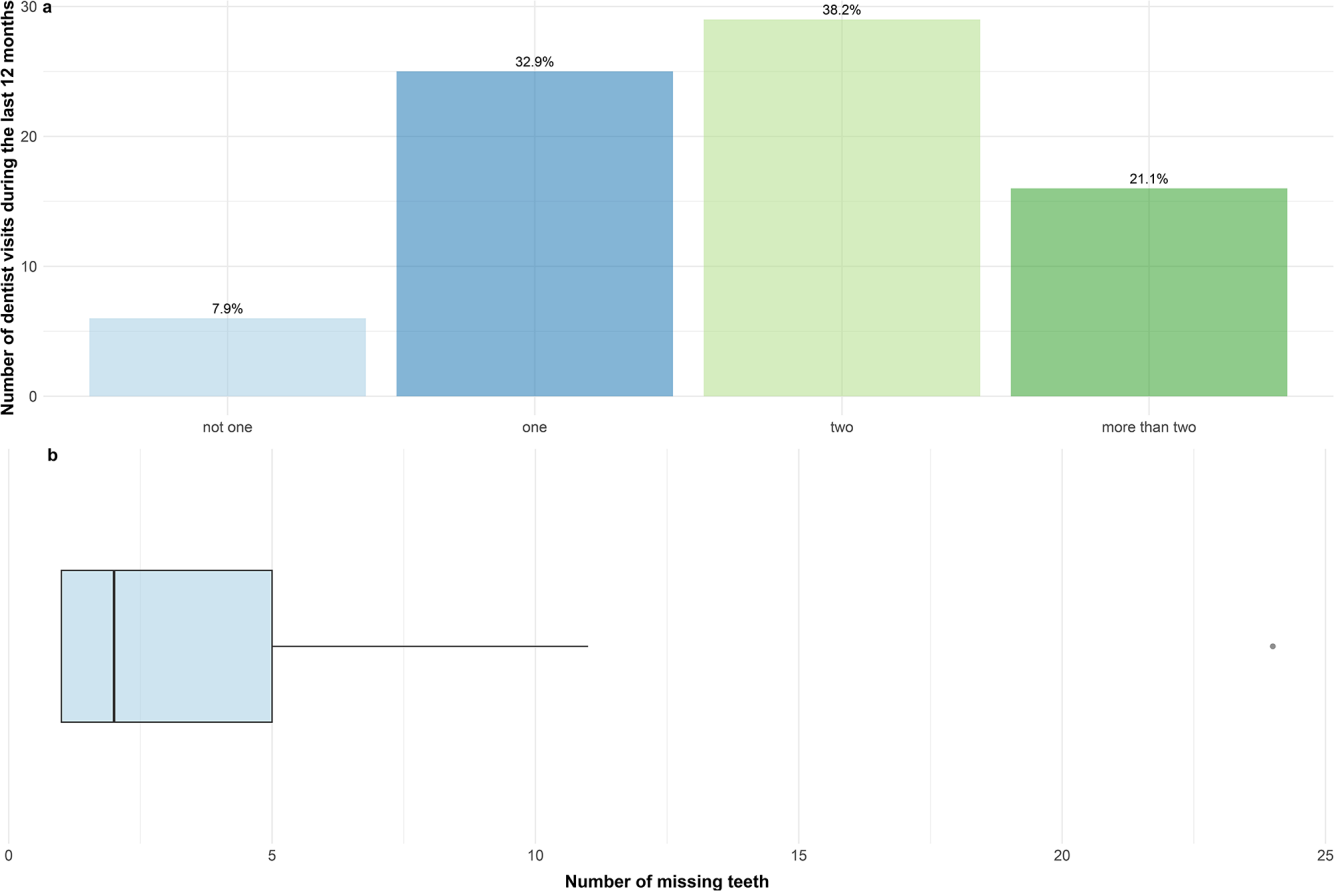
Oral health data Part **a** shows the number of dental visits in the last 12 months. Part **b** shows the number of missing teeth

**Fig. 2:**
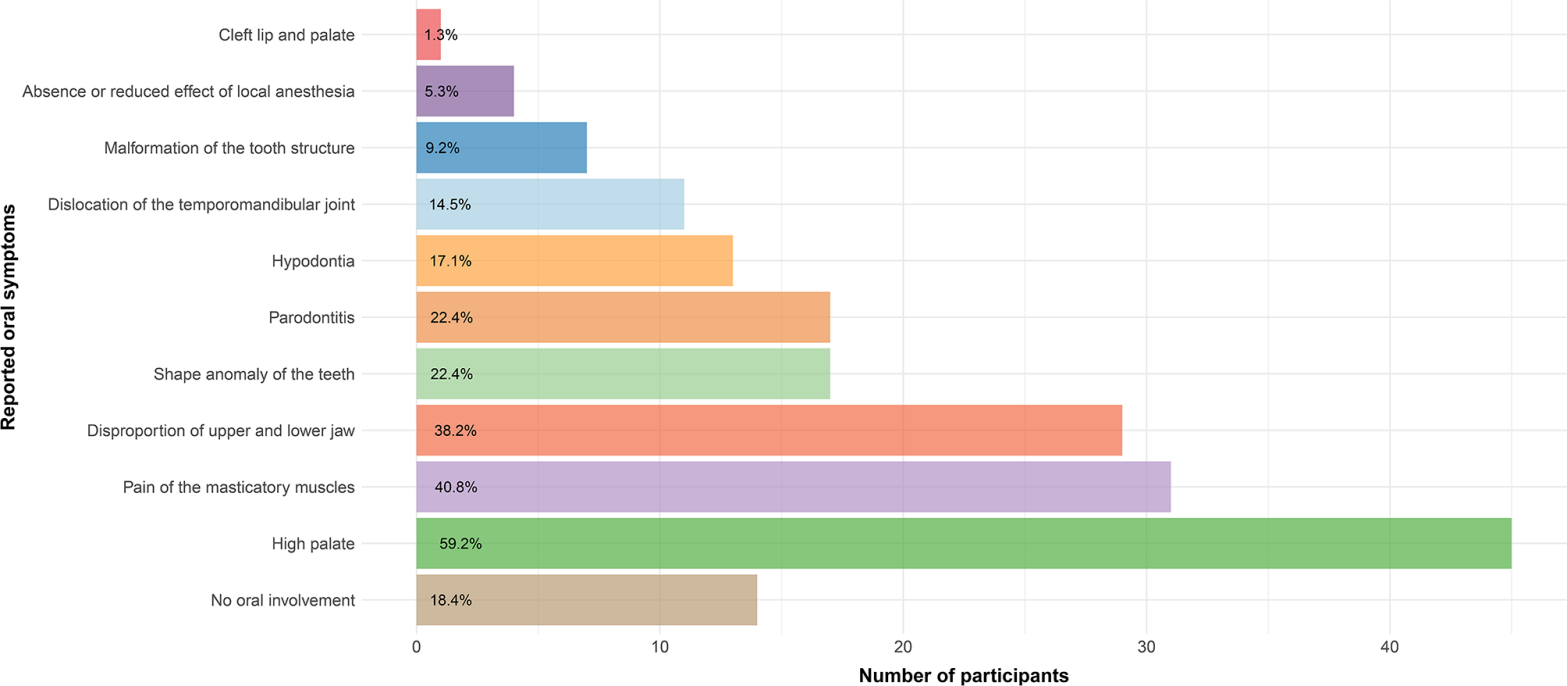
Oral symptoms Reported oral symptoms in participants with marfan syndrome

Only 7.9% of the participants reported not having visited the dentist during the last 12 months. Reported oral symptoms and oral cavity involvement varied widely, with 81.6% reporting oral involvement. The most prevalent described symptoms were “High palate” (59.2%), “clenching or grinding the teeth” (52.6%), “Pain in the masticatory muscles” (40.8%), and “Disproportion of upper and lower jaw” (38.2%). An uneven first contact when biting down was reported by 24 participants(31.6%) of participants. Detailed information regarding oral symptoms can be seen in Figs. 1 and 2.

### TMD-specific symptoms

50 participants (65.8%) showed TMD symptoms. Within pain or restrictions when moving the jaw, chewing was reported to cause pain most frequently, with 39.5%. Regarding the masticatory muscles, pain most frequently occurred in the region of the jaw angle (50.0%). 42.1% of participants had no pain in the masticatory muscles. Most participants reported having a symmetrical mouth opening (73.7%). Detailed information regarding TMD symptoms can be seen in Table 2.

### Diagnosed TMD and treatment

A TMD was already diagnosed in eleven (14,5%) patients. At the time of the study, five (45,5%) of them received a TMD-specific treatment, including physiotherapy (*n* = 3) and an occlusal splint (*n* = 4). Three out of five reported no change in the situation with the treatment received. One participant felt a slight, and one participant felt a significant improvement. Detailed information regarding diagnosed TMD symptoms can be seen in Table 3.

**Table 2:** Specific temporomandibular disorders (TMD) symptoms in participants with marfan syndrome

TMD symptoms	*n*	%
**Painful or restricted movements of the jaw**		
no painful or restricted movements	34	44.7
while chewing	30	39.5
while mouth opening	21	27.6
while mouth closing	6	7.9
**Pain or stiffness of the masticatory muscles**		
no pain or stiffness	32	42.1
pain in the buccal area	15	19.7
pain in the jaw angle	38	50.0
pain in the temporal region	19	25
symmetrical mouth opening	56	73.3

**Table 3:** Symptoms in participants diagnosed with temporomandibular disorders (*n* = 11)

	*n*	%
Cracking or rubbing of the left temporomandibular joint	7	63.6
Cracking or rubbing of the right temporomandibular joint	5	45.5
Pain in the masticatory muscles	7	63.6
Pain in the muscles of the neck	9	81.8

### Evaluation of the DASS

There were missing data in five cases. Seventy-one questionnaires were included for analysis. The evaluation revealed that 58 participants (81.7%) are below the threshold value for depression. In comparison, 13 participants (18.3%) reached the critical value of ten and have an increased likelihood of a depressive disorder. The critical value of 6 on the anxiety scale was reached by 22 participants (31.0%), with 69.0% [49] below that threshold value. On the stress scale, there were 56 participants (78.9%) below the threshold value and 15 participants (21.1%) reaching the critical value of 10.

A noticeable difference was found between participants with and without TMD symptoms and the increased likelihood of an anxiety disorder (*p* = < 0.001). The majority of participants had TMD symptoms that exceeded the threshold for increased likelihood of occurrence of depression (score > 10), anxiety (score > 6), or stress disorder (score > 10). Detailed information for all three categories can be seen in Table 4.

**Table 4:** Number of participants with increased likelihood of depression/anxiety/stress according to the threshold values of the Depression Anxiety Stress Scale (DASS)

DASS	TMD symptoms (*n* = 45)	no TMD symptoms (*n* = 26)	*p*-value
depression (*n* = 13)	10 (76.9%)	3 (23.1%)	0.262
no depression (*n* = 58)	35 (60.3%)	23 (39.7%)	
anxiety (*n* = 22)	22 (90.9%)	0 (9.1%)	< 0.001
no anxiety (*n* = 49)	26 (53.1%)	23 (46.9%)	
stress (*n* = 15)	11 (73.3%)	4 (26.7%)	0.435
no stress (*n* = 56)	34 (60.7%)	22 (39.3%)	

Number of participants divided according to the presence of symptoms of temporomandibular disorders (TMD). Line percentages are given. (No) depression, anxiety, and stress are based on whether the score of the DASS was below or above the threshold value for the individual category

### Evaluation of the GCS

The evaluation of the GCS revealed that 27 participants (35.5%) had no pain (grade 0), 39 participants (51.3%) had low impairment with low pain intensity (grade 1), four participants (5.3%) had low impairment with high pain intensity (grade 2), five participants (6.6%) had severe impairment with moderate limitations (grade 3), and one participant (1.3%) had severe impairment with high limitations (grade 4).

According to the GCS, none of the participants without TMD symptoms are graded higher than “low impairment – low pain intensity” (grade 1). Most of those participants were graded as having “no pain” (81.5%). A noticeable difference between the groups “TMD symptoms” and “no TMD symptoms” was observed (*p* = < 0.001). Detailed information are presented in Table 5.

**Table 5:** Number of participants with chronic pain according to the questionnaire “Graduierung chronischer Schmerzen” (GCS) concerning the presence of symptoms of temporomandibular disorders

Grades according to GCS	*n*	TMD symptoms (*n* = 50)	no TMD symptoms (*n* = 26)	*p*-value
no pain	27 (35.5%)	5 (18.5%)	22 (81.5%)	*< 0.001*
low impairment - low pain intensity	39 (51.3%)	35 (89.7%)	4 (10.2%)	
*low impairment - high pain intensity*	*4 (5.3%)*	*4 (100.0%)*	*0 (0.0%)*	
*severe impairment - moderate limitation*	*5 (6.6%)*	*5 (100.0%)*	*0 (0.0%)*	
*severe impairment - high limitation*	*1 (1.3%)*	*1 (100.0%)*	*0 (0.0%)*	

### OHRQoL

The median OHIP-G14 score was 6 (IQR 15). Specific questions were answered often with a noticeably high score. Nine participants (11.8%) reported that their life in general “often” or “very often” was less satisfying. One in four participants indicated that they “often” or “very often” had problems with their teeth, mouth, or dentures in the past month, making it difficult to relax. 21.1% of the people [16] reported feeling tense “often” or “very often” in the past month. The question of whether there was any pain in the mouth area in the past month was answered with “often” or “very often” by 16 participants (21.1%). Detailed information about each item can be seen in Supplementary File 2. The calculated scores of the four dimensions of OHRQoL are presented in Table 6. In each of the four dimensions, the scores for participants with TMD symptoms were noticeably higher than the scores for participants without TMD symptoms. Orofacial appearance had the highest score (2.29 ±2.51), and Oral function seemed to cause the slightest impairment in our cohort (0.97 ±1.60). In the median, participants with TMD symptoms had notably higher scores compared to those without TMD symptoms (11.5 [IQR 17] vs. 1 [IQR 3], *p* = < 0.001).

**Table 6:** Four dimensions of oral health-related quality of life (OHRQoL). Scores are given individually for the group with and without TMD symptoms, together with a total score

Dimensions of OHRQoL	TMD symptoms	no TMD symptoms	total	*p*-value
Oral Function	1.44 (±1.80)	0.08 (±0.27)	0.97 (±1.60)	< 0.001
Orofacial Pain	3.14 (±2.37)	0.27 (±0.53)	2.16 (±2.38)	< 0.001
Orofacial Appearance	3.18 (±2.60)	0.58 (±1.03)	2.29 (±2.51)	< 0.001
Psychosocial Impairment	1.38 (±1.71)	0.23 (±0.43)	0.99 (±1.51)	0.002

#### Psychological impairment and OHRQoL

**Fig. 3:**
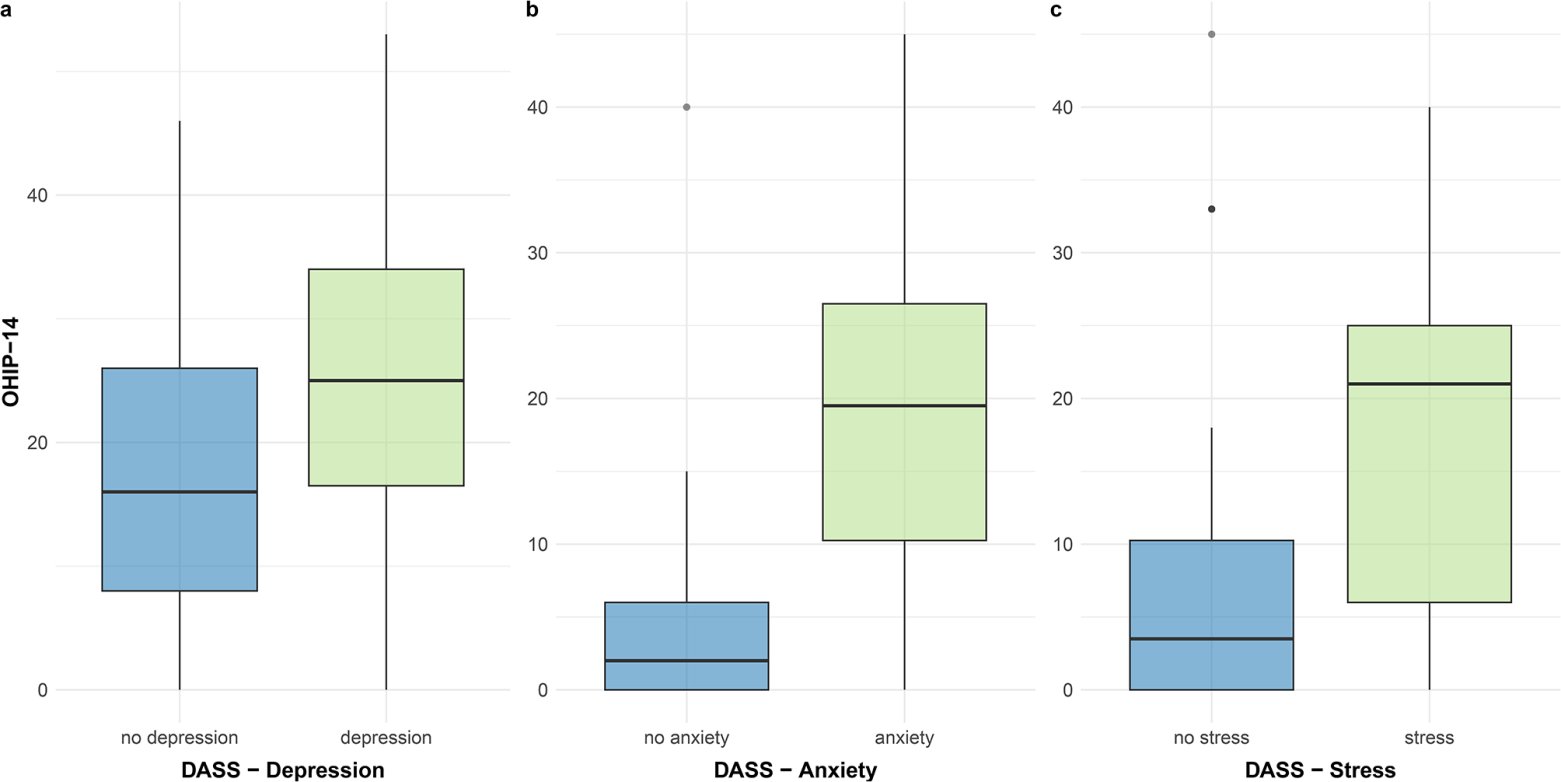
Psychological Impairment and oral health-related quality of life Parts **a**-**c**: German short form of the Oral Health Impact Profile (OHIP-G14) depending on the Depression Anxiety Stress Scale (DASS) categories

Figure 3 shows the difference in OHIP-G14 for the three DASS categories depression, anxiety, and stress. Participants with an increased likelihood of any of these disorders showed an increased OHIP-G14 value. The median in the two groups in the depression category differs noticeably by 16 points (*p* = 0.017). In the anxiety disorder category, the difference was 17.5 points (*p* = < 0.001), and in the stress category, by 17.5 points (*p* = 0.005).

#### Chronic pain and OHRQoL

**Fig. 4:**
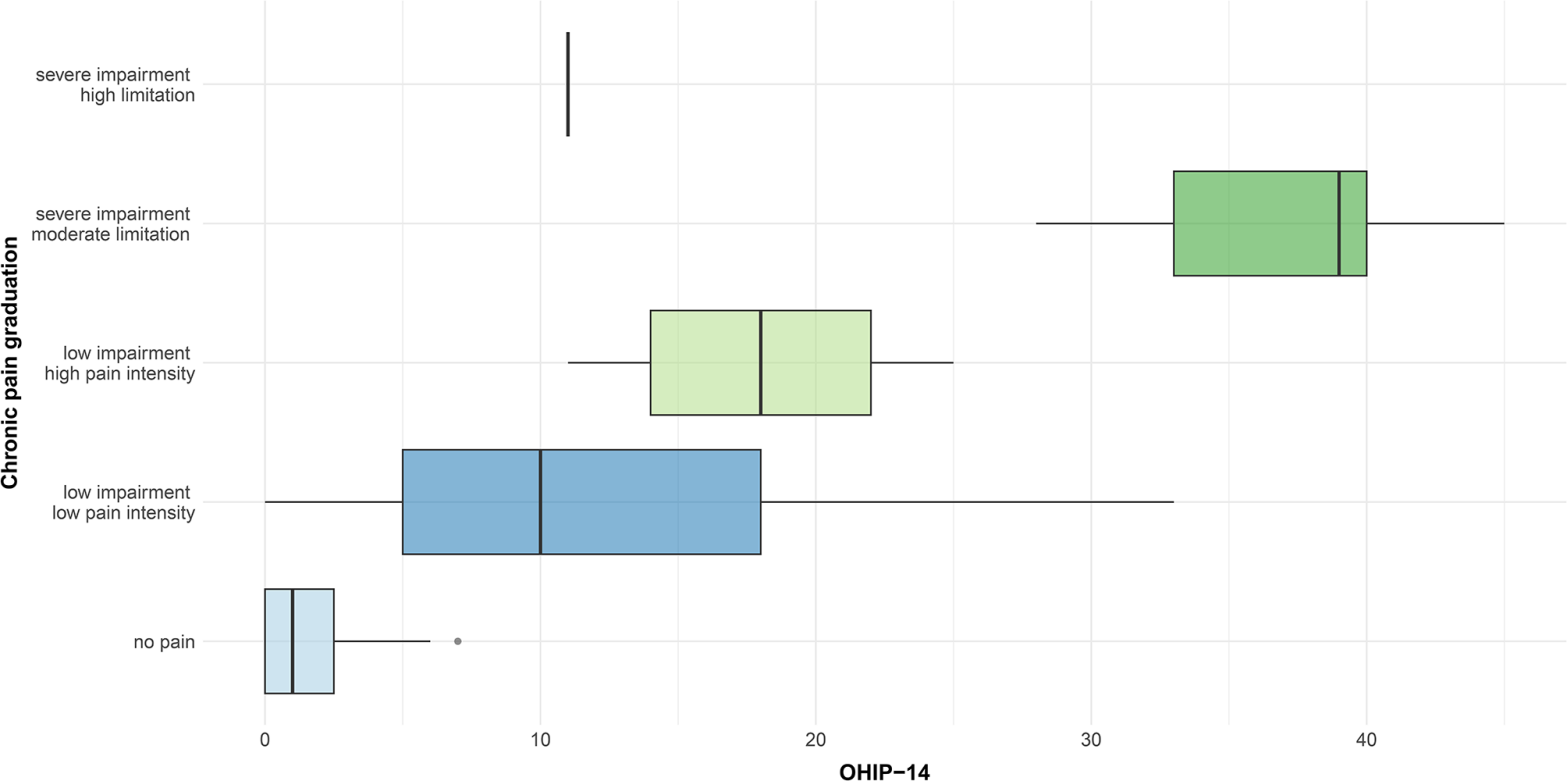
Chronic Pain and oral health-related quality of life *Information about the different grades of the questionnaire “Graduierung chronischer Schmerzen” (GCS) concerning the German short form of the Oral Health Impact Profile (OHIP-G14).*

A noticeable difference was observed between the five grades of the GCS and the OHIP-G14 (*p* = < 0.001). The most severe impact on the OHRQoL could be seen in participants graded with “severe impairment – moderate limitation,” with an OHIP-G14 score 38 points higher than participants graded with “no pain”. Boxplots for all five grades are presented in Fig. 4.

## Discussion

This study aimed to analyze to what extent patients with Marfan syndrome are affected by TMD, especially the impact on oral health-related quality of life. A high number of participants in this study showed typical symptoms that occur in TMD. Over half of the participants named clenching or grinding with their teeth or pain in at least one of the masticatory muscles as one of their symptoms. These symptoms may be related to joint hypermobility similar to Ehlers-Danlos syndromes, in which some subtypes also show a high prevalence of TMD symptoms [10, 11, 21–23]. Previous studies have shown similar results for patients with Marfan syndrome experiencing TMD-like symptoms. For example, de Coster et al. studied a cohort of 42 individuals with generalized joint hypermobility (GJH), of which 24 were diagnosed with Marfan syndrome. A positive correlation between GJH and TMD was shown, and 71.4% of all participants showed signs of TMD [11]. This is in accordance with Bauss et al., who surveyed 281 individuals with Marfan syndrome and concluded that TMD is a critical aspect in affected individuals, with over half of the participants showing symptoms of TMD [14]. Furthermore, Staufenbiel et al. concluded that the prevalence of temporomandibular joint disorders seems to be higher in Marfan patients than in patients without MFS [24]. This prevalence of TMD can be seen as reasonably high compared to the general population, which ranges from 15 to 31%, according to multiple studies [25, 26]. However, only 14.5% of participants in our cohort had already been diagnosed with TMD. This low number seems underdiagnosed since more participants showed TMD-like symptoms (65.8%). This suggests that greater focus should be placed on the diagnosis of TMD in individuals with Marfan syndrome to provide adequate therapy.

One of the main symptoms of TMD is pain in the orofacial region. In our cohort, most participants reported pain ranging from low impairment and intensity to severe impairment and intensity. According to a systematic review of chronic pain in MFS, the prevalence of chronic pain ranged from 47–92% [27]. Pain is considered chronic if it persists longer than 3 to 6 months or recurs [28]. Only one study has focused on pain in the temporomandibular region [14]. Results from this study are comparable to ours, as more than half of the participants reported pain in the TMJ area. The assessment of chronic pain in our cohort showed an increase in pain severity with the presence of TMD symptoms. None of the participants without TMD symptoms showed dysfunctional chronic pain on the chronic pain assessment. Pain in its chronic form can cause various problems, including “chronic fatigue, sleep disturbances, excessive rest, withdrawal from activity, reduced sexual activity, compromised immune function and mood disorders” [27, 29]. Speed et al. concluded that MFS should be regarded as a chronic pain disorder and can lead to “profound disability and psychological burden” [30].

The results of our study suggest this, as a considerable number of participants showed an increased susceptibility to depression, anxiety or stress disorders (18.3%, 31.0%, and 21.1%, respectively). Peters et al. also reported significant depressive symptoms in over 40% of an MFS cohort [31]. The results of other studies showed similar impairments in MFS regarding anxiety, depression, mental fatigue, and other psychiatric symptoms [30, 32–36]. Our data show that TMD symptoms may be associated with psychological impairment. It is essential that the psychological findings are taken seriously but that this does not lead to a psychiatric misdiagnosis. Patients with Marfan syndrome who initially received a psychiatric diagnosis waited an average of 14 years until the correct diagnosis of Marfan syndrome was made [37]. To date, no comparable studies address the impact of TMD on psychological impairment in individuals with MFS. However, Yap et al. reported that “Participants with […] TMD symptoms generally exhibited significantly higher levels of psychological distress and worse OHRQoL” [38].

Previous studies have shown the impact of MFS and its psychological impairment on Quality of Life (QoL) [33–36, 39–41]. However, only one study investigated the OHRQoL in people with MFS in Germany, and the results suggested that people with MFS show a reduced OHRQoL (OHIP-G14: 13.65 ± 13.53 ) compared to the general population [6]. When considering the median OHIP value in our cohort, the score was comparably better (median of 6 (IQR 15)). However, this value also showed a reduced OHRQoL compared to the German general population. John et al. observed that 80% of the naturally toothed participants in a representative study had an OHIP-G14 value of ≤ six [42].

The OHRQoL of people with MFS was reduced in our study, with the median OHIP of people with TMD symptoms being 10.5 points higher than that of people without these symptoms. Similar results were reported by Hanna et al. [43]. A reduced OHRQoL was observed in their cohort of Australian adults who reported TMD experience. To better assess the extent of the eight-point difference in our cohort, one can refer to results from a study by Reissmann et al. For the OHIP-G14, one point difference was associated with nearly two impacts on OHRQoL per day [44]. Reissmann et al. found that individuals with TMD had at least three times the impact on OHRQoL as individuals without TMD [44, 45]. This considerably high impact of TMD on OHRQoL is supported by the results of several studies [46–49]. In a case-control study by Bayat et al., the most important predictors of OHRQoL were an increased level of chronic pain and psychological impairment [50]. With increasing levels of chronic pain, participants in our cohort also showed a reduction in OHRQoL. Noticeable higher values for the dimension of Oral Function, Orofacial Pain, and Orofacial appearance were observed for the group with TMD symptoms. The presence of symptoms indicating depression, anxiety or stress disorder also led to a reduced OHRQoL.

The EurordisCare surveys have shown that the need for dental care (64%) is very high in people with Marfan syndrome [37]. Based on this and the findings of our study, several approaches seem to be needed to improve the oral health and related impairments of people affected by MFS. A vital role in this context should be the diagnosis and adequate treatment of TMD to improve OHRQoL. Our data may indicate that the occurrence of TMD in MFS may be overlooked to a considerable extent. Only five of the already diagnosed participants reported being in TMD-specific treatment, of which only two felt an improvement. No studies available demonstrate the potential effects of TMD-specific therapy in MFS. In the general population, a combination of conservative therapies relieved pain in 50–90% of patients [26]. Therefore, it seems necessary to investigate whether targeted non-invasive therapies can improve the situation. Several studies have recommended using non-invasive therapies such as pharmacotherapy, physiotherapy, and occlusal splints. Self-care, patient education, and cognitive behavior therapy should also not be neglected [21, 26, 51].

### Limitations

One limitation of this study is the small sample size; however, MFS is a rare disease that is not frequently encountered. Within participants, there was a considerable gender imbalance, with over 80% of participants being female. However, in a cohort of participants affected by rare diseases, Bohner et al. showed no impact of gender on OHRQoL [52]. In addition, the data were collected using an online questionnaire, meaning only the participants’ subjective assessments can be reported. As this is a cross-sectional study, the results should be interpreted with caution and no causal conclusions should be drawn. This would require further case-control studies. Clinical studies with more participants are also needed to verify these subjective findings.

## Conclusions

This study showed that TMD symptoms were common in our cohort of individuals with MFS and were associated with a reduced OHRQoL. Participants with an increased level of chronic pain and psychological impairment showed a greater reduction of OHRQoL. TMD seemed underdiagnosed in MFS since far more participants had TMD symptoms than those reported having a TMD diagnosis. Further studies should emphasize the diagnosis and treatment of TMD. Our data suggest that early and adequate therapy seemed necessary to prevent the chronification of pain and associated psychological burden and thus improve the OHRQoL.

### Electronic supplementary material

Below is the link to the electronic supplementary material.

Supplementary Material 1: Supplementary File 1 contains the free-text questions from the online questionnaire. The questionnaire was initially designed in German. Supplementary File 1 is a translation of the german questionnaire

## Data Availability

All data generated or analyzed during this study are included in this published article (and its supplementary information files). The datasets used and analyzed during the current study are available from the corresponding author upon reasonable request.

## Declarations

### Additional files

Supplementary File 1 contains the free-text questions from the online questionnaire. The questionnaire was initially designed in German. Supplementary File 1 is a translation of the german questionnaire.

## Abbreviations

MFS: Marfan syndrome
TMD: temporomandibular disorders
OHRQoL: oral health-related quality of life
OHIP-G14: Oral Health Impact Profile
DASS: Depression Anxiety Stress Scale
GCPS: Grades Chronic Pain Status
FBN1: fibrillin-1 gene
GJH: generalized joint hypermobility
EDS: Ehlers-Danlos Syndrome
IQR: interquartile range
GCS: Graduierung chronischer Schmerzen

## References

[1] Judge DP, Dietz HC (2005). Marfan’s syndrome. Lancet.

[2] Sivasankari T, Mathew P, Austin RD, Devi S (2017). Marfan Syndrome. J Pharm Bioallied Sci.

[3] Yuan SM, Jing H (2010). Marfan’s syndrome: an overview. Sao Paulo Med J.

[4] Milewicz DM, Braverman AC, De Backer J, Morris SA, Boileau C, Maumenee IH (2021). Marfan syndrome. Nat Rev Dis Primers.

[5] Cañadas V, Vilacosta I, Bruna I, Fuster V (2010). Marfan syndrome. Part 1: pathophysiology and diagnosis. Nat Rev Cardiol.

[6] Hanisch M, Wiemann S, Jung S, Kleinheinz J, Bohner L. Oral health-related quality of life in people with Rare Hereditary Connective tissue disorders: Marfan Syndrome. Int J Environ Res Public Health. 2018;15(11). 10.3390/ijerph15112382 PMC6266687 30373236

[7] Westling L, Mohlin B, Bresin A (1998). Craniofacial manifestations in the Marfan syndrome: palatal dimensions and a comparative cephalometric analysis. J Craniofac Genet Dev Biol.

[8] Cervino G, Cicciù M, De Stefano R, Falcomatà D, Bianchi A, Crimi S (2020). Oral health in patients with Marfan syndrome. Arch Oral Biol.

[9] Buescher JJ (2007). Temporomandibular joint disorders. Am Fam Physician.

[10] Hirsch C, John MT, Stang A (2008). Association between generalized joint hypermobility and signs and diagnoses of temporomandibular disorders. Eur J Oral Sci.

[11] De Coster PJ, Van den Berghe LI, Martens LC (2005). Generalized joint hypermobility and temporomandibular disorders: inherited connective tissue disease as a model with maximum expression. J Orofac Pain.

[12] Qamar Z, Alghamdi AMS, Haydarah NKB, Balateef AA, Alamoudi AA, Abumismar MA (2023). Impact of temporomandibular disorders on oral health-related quality of life: a systematic review and meta‐analysis. J Oral Rehabilitation.

[13] Ujin Yap A, Cao Y, Zhang M, Lei J, Fu K (2021). Age-related differences in diagnostic categories, psychological states and oral health–related quality of life of adult temporomandibular disorder patients. J Oral Rehabilitation.

[14] Bauss O, Sadat-Khonsari R, Fenske C, Engelke W, Schwestka-Polly R (2004). Temporomandibular joint dysfunction in Marfan syndrome. Oral Surg Oral Med Oral Pathol Oral Radiol Endod.

[15] John MT, Miglioretti DL, LeResche L, Koepsell TD, Hujoel P, Micheelis W (2006). German short forms of the oral Health Impact Profile. Commun Dent Oral Epidemiol.

[16] Nilges P, Essau CDASS. Depressions-Angst-Stress-Skalen - deutschsprachige Kurzfassung. 2021 May [cited 2022 Jun 20]; https://www.psycharchives.org/handle/20.500.12034/4089

[17] Von Korff M, Ormel J, Keefe FJ, Dworkin SF (1992). Grading the severity of chronic pain. Pain.

[18] Türp J, Nilges P (2000). Diagnostik Von Patienten Mit Chronischen Orofazialen Schmerzen. Die deutsche Version Des „Graded Chronic Pain Status. Quintessenz.

[19] Schiffman E, Ohrbach R, Truelove E, Look J, Anderson G, Goulet JP (2014). Diagnostic criteria for Temporomandibular disorders (DC/TMD) for clinical and Research Applications: recommendations of the International RDC/TMD Consortium Network* and Orofacial Pain Special Interest Group†. J Oral Facial Pain Headache.

[20] John M, Omara M, Su N, List T, Sekulic S, Häggman-Henrikson B et al. RECOMMENDATIONS FOR USE AND SCORING OF ORAL HEALTH IMPACT PROFILE VERSIONS. J Evid Based Dent Pract. 2021;101619. 10.1016/j.jebdp.2021.101619 PMC8886153 35219460

[21] Mitakides J, Tinkle BT (2017). Oral and mandibular manifestations in the Ehlers-Danlos syndromes. Am J Med Genet.

[22] Diep D, Fau V, Wdowik S, Bienvenu B, Bénateau H, Veyssière A (2016). [Temporomandibular disorders and Ehlers-Danlos syndrome, hypermobility type: a case-control study]. Rev Stomatol Chir Maxillofac Chir Orale.

[23] Norton LA, Assael LA (1997). Orthodontic and temporomandibular joint considerations in treatment of patients with Ehlers-Danlos syndrome. Am J Orthod Dentofac Orthop.

[24] Staufenbiel I, Hauschild C, Kahl-Nieke B, Vahle-Hinz E, von Kodolitsch Y, Berner M (2013). Periodontal conditions in patients with Marfan syndrome - a multicenter case control study. BMC Oral Health.

[25] Valesan LF, Da-Cas CD, Réus JC, Denardin ACS, Garanhani RR, Bonotto D (2021). Prevalence of temporomandibular joint disorders: a systematic review and meta-analysis. Clin Oral Invest.

[26] Gauer RL, Semidey MJ (2015). Diagnosis and treatment of temporomandibular disorders. Am Fam Physician.

[27] Velvin G, Bathen T, Rand-Hendriksen S, Geirdal AØ (2016). Systematic review of chronic pain in persons with Marfan syndrome: systematic review, Marfan syndrome, chronic pain, associated factors. Clin Genet.

[28] Treede RD, Rief W, Barke A, Aziz Q, Bennett MI, Benoliel R (2015). A classification of chronic pain for ICD-11. Pain.

[29] Chapman CR, Gavrin J (1999). Suffering: the contributions of persistent pain. Lancet.

[30] Speed TJ, Mathur VA, Hand M, Christensen B, Sponseller PD, Williams KA (2017). Characterization of pain, disability, and psychological burden in Marfan syndrome. Am J Med Genet A.

[31] Peters K, Kong F, Horne R, Francomano C, Biesecker B (2001). Living with Marfan syndrome I. perceptions of the condition: perceptions of Marfan syndrome. Clin Genet.

[32] Andonian C, Freilinger S, Achenbach S, Ewert P, Gundlach U, Kaemmerer H (2021). Quality of life in patients with Marfan syndrome: a cross-sectional study of 102 adult patients. Cardiovasc Diagn Ther.

[33] Ratiu I, Virden TB, Baylow H, Flint M, Esfandiarei M (2018). Executive function and quality of life in individuals with Marfan syndrome. Qual Life Res.

[34] Fusar-Poli P, Klersy C, Stramesi F, Callegari A, Arbustini E, Politi P (2008). Determinants of quality of life in Marfan Syndrome. Psychosomatics.

[35] Rand-Hendriksen S, Sørensen I, Holmström H, Andersson S, Finset A (2007). Fatigue, cognitive functioning and psychological distress in Marfan syndrome, a pilot study. Psychol Health Med.

[36] Nielsen C, Ratiu I, Esfandiarei M, Chen A, Selamet Tierney ES (2019). A review of psychosocial factors of Marfan Syndrome: adolescents, adults, families, and providers. J Pediatr Genet.

[37] EURORDIS AKFF. The Voice of 12,000 Patients. Experiences and Expectations of Rare Disease Patients on Diagnosis and Care in Europe [Internet]. Eurordis. 2009. https://www.eurordis.org/wp-content/uploads/2009/12/EURORDISCARE_FULLBOOKr.pdf

[38] Yap AU, Cao Y, Zhang MJ, Lei J, Fu KY (2021). Number and type of temporomandibular disorder symptoms: their associations with psychological distress and oral health-related quality of life. Oral Surg Oral Med Oral Pathol Oral Radiol.

[39] Moon JR, Cho YA, Huh J, Kang IS, Kim DK (2016). Structural equation modeling of the quality of life for patients with marfan syndrome. Health Qual Life Outcomes.

[40] Gritti A, Pisano S, Catone G, Iuliano R, Salvati T, Gritti P (2015). Psychiatric and neuropsychological issues in Marfan syndrome: a critical review of the literature. Int J Psychiatry Med.

[41] Trawicka A, Lewandowska-Walter A, Majkowicz M, Sabiniewicz R, Woźniak-Mielczarek L (2022). Health-related quality of life of patients with Marfan Syndrome-Polish Study. Int J Environ Res Public Health.

[42] John MT, Micheelis W, Biffar R (2004). [Reference values in oral health-related quality of life for the abbreviated version of the oral Health Impact Profile]. Schweiz Monatsschr Zahnmed.

[43] Hanna K, Nair R, Amarasena N, Armfield JM, Brennan DS (2021). Temporomandibular dysfunction experience is associated with oral health-related quality of life: an Australian national study. BMC Oral Health.

[44] Reissmann DR, Sierwald I, Heydecke G, John MT (2013). Interpreting one oral health impact profile point. Health Qual Life Outcomes.

[45] Reißmann DR, John MT, Schierz O, Wassell RW (2007). Functional and psychosocial impact related to specific temporomandibular disorder diagnoses. J Dent.

[46] Dasukil S, Arora G, Shetty S, Degala S (2021). Impact of prolotherapy in temporomandibular joint disorder: a quality of life assessment. Br J Oral Maxillofac Surg.

[47] Almoznino G, Zini A, Zakuto A, Sharav Y, Haviv Y, Avraham H (2015). Oral health-related quality of life in patients with Temporomandibular disorders. J Oral Facial Pain Headache.

[48] Lei J, Yap AU, Zhang M, Fu K (2021). Temporomandibular disorder subtypes, emotional distress, impaired sleep, and oral health-related quality of life in Asian patients. Community Dent Oral Epidemiol.

[49] John MT, Reissmann DR, Schierz O, Wassell RW (2007). Oral health-related quality of life in patients with temporomandibular disorders. J Orofac Pain.

[50] Bayat M, Abbasi A, Noorbala A, Mohebbi S, Moharrami M, Yekaninejad M (2018). Oral health-related quality of life in patients with temporomandibular disorders: a case-control study considering psychological aspects. Int J Dent Hygiene.

[51] Wieckiewicz M, Boening K, Wiland P, Shiau YY, Paradowska-Stolarz A (2015). Reported concepts for the treatment modalities and pain management of temporomandibular disorders. J Headache Pain.

[52] Bohner L, Wiemann S, Jung S, Kleinheinz J, Hanisch M (2019). Oral health-related quality of life in rare diseases associated with oral symptoms, diagnostic delay, and sex.

