# Supplementary material for: Temporomandibular disorders in individuals with Marfan syndrome: an exploratory analysis
Source: Head Face Med. 2024 Apr 24;20:26. doi: 10.1186/s13005-024-00427-z (PMC11040890; doi:10.1186/s13005-024-00427-z)
Supplement: Supplementary file 1 — Supplementary Material 1: Supplementary File 1 contains the free-text questions from the online questionnaire. The questionnaire was initially designed in German. Supplementary File 1 is a translation of the german questionnaire [file 13005_2024_427_MOESM1_ESM.docx]

# Supplementary File S1

# Online Questionaire

1. **Sex**

- Male
- Female

1. **Age**

*The age was entered as free text.*

1. **In which country do you live**

- Germany
- Austria
- Switzerland

1. **How old were you when you were diagnosed with your disease?**

*The age was entered as free text.*

1. **How much time has elapsed between the first appearance of symptoms and the definitive diagnosis of your disease?**

*The years were given as free text.*

1. **How many teeth have you already lost (without wisdom teeth)?**

*The number of teeth was given as free text.*

1. **How often have you seen a dentist within the last 12 months?**

- Not once
- Once
- Twice
- More often than twice

1. **Does your condition involve any of the following involvement of the oral cavity or face?**

- Absence or reduced effect of local anesthesia
- Pain of the masticatory muscles
- Parodontitis
- Hypodontia
- Disproportion of upper and lower jaw
- Cleft lip and palate
- High palate
- Shape anomaly of the teeth
- Malformation of the tooth structure
- Dislocation of the temporomandibular joint
- No oral involvement

1. **Are there currently, or have there been in the past, one or more of the following movements that are limited and/or painful?**
   **(Multiple answers are possible)**

- No
- Chewing
- Mouth opening
- Mouth closure
- Lateral jaw movements

1. **When you open your mouth slowly, does it open straight (symmetrical) or does it deviate to one side (asymmetrical)?**
   **Please use a mirror for this purpose or have someone else examine the mouth opening. Pay particular attention to the movement of the incisors and repeat the opening and closing movement several times.**

- Not feasible
- Symmetrical
- Asymmetrical

1. **Do you currently have, or have you had in the past, hardening and/or pain of the muscles in one or more of the following locations?**
   **(Multiple answers are possible)**

- No pain or hardening
- Cheek
- Jaw angel
- Temple

1. **When you bite down slowly and in a controlled manner, do you feel that your upper and lower molars meet at the same time or do individual teeth touch first before full contact can be made?**

- Rather simultaneous
- Rather first individual teeth

1. **Do you clench your teeth and/or grind them during the day and/or during the night? Have you been made aware of this by sleep partners, if applicable?**

- Yes
- No
- I don’t know

1. **Have you been diagnosed with temporomandibular dysfunction (TMD)?**
   **Temporomandibular dysfunction, or TMD for short, is the term used to describe a painful dysregulation of the masticatory system.**

**It can be triggered by a disturbed interaction of various muscles and joints.**

- Yes
- No

*The following question “15.” could only be answered if the previous question was answered with Yes.*

1. **What TMD-specific symptoms have you been diagnosed with?**
   **(Multiple answers are possible)**

- Cracking/grating of the right temporomandibular joint
- Cracking/grating of the left temporomandibular joint
- Pain of the masticatory muscles
- Pain of the neck muscles
- Other symptoms

1. **Are you currently receiving treatment for CMD?**
   **e.g. in the form of splint therapy, physiotherapy etc.**

- Yes
- No

*The following 2 questions “17.” and “18.” could only be answered if the previous question was answered with Yes.*

1. **In what form is CMD currently being treated?**
   **(Multiple answers are possible)**

- Splint therapy
- Physiotherapy
- Acupuncture
- Behavioral therapy
- Ergotherapy
- Other type of treatment

1. **Do you feel any improvement as a result of the treatment?**

- Yes, a significant improvement
- Yes, a slight improvement
- I do not feel any difference
- No, I feel a worsening
